# Mortality and suicide in schizophrenia: 21-year follow-up in rural China

**DOI:** 10.1192/bjo.2020.106

**Published:** 2020-10-15

**Authors:** Mao-Sheng Ran, Yunyu Xiao, Seena Fazel, Yeonjin Lee, Wei Luo, Shi-Hui Hu, Xin Yang, Bo Liu, Maria Brink, Sherry Kit Wa Chan, Eric Yu-Hai Chen, Cecilia Lai-Wan Chan

**Affiliations:** Department of Social Work and Social Administration, University of Hong Kong, China; School of Social Work, Indiana University–Bloomington and IUPUI, Indianapolis, USA; Department of Psychiatry, Warneford Hospital, University of Oxford, UK; Department of Social Work and Social Administration, University of Hong Kong, China; Xinjin Second People's Hospital, Chengdu, China; Chengdu Mental Health Center, China; Guangyuan Mental Health Center, China; Jingzhou Mental Health Center, China; Department of Psychiatry, University of Rochester Medical Center, New York, USA; Department of Psychiatry, University of Hong Kong, China; Department of Psychiatry, University of Hong Kong, China; Department of Social Work and Social Administration, University of Hong Kong, China

**Keywords:** Suicide, mortality, schizophrenia, China, predictive factors

## Abstract

**Background:**

Little is known about the trend and predictors of 21-year mortality and suicide patterns in persons with schizophrenia.

**Aims:**

To explore the trend and predictors of 21-year mortality and suicide in persons with schizophrenia in rural China.

**Method:**

This longitudinal follow-up study included 510 persons with schizophrenia who were identified in a mental health survey of individuals (≥15 years old) in 1994 in six townships of Xinjin County, Chengdu, China, and followed up in three waves until 2015. Kaplan–Meier survival analysis and Cox hazard regressions were conducted.

**Results:**

Of the 510 participants, 196 died (38.4% mortality) between 1994 and 2015; 13.8% of the deaths (*n* = 27) were due to suicide. Life expectancy was lower for men than for women (50.6 *v*. 58.5 years). Males consistently showed higher rates of mortality and suicide than females. Older participants had higher mortality (hazard ratio HR = 1.03, 95% CI 1.01–1.05) but lower suicide rates (HR = 0.95, 95% CI 0.93–0.98) than their younger counterparts. Poor family attitudes were associated with all-cause mortality and death due to other causes; no previous hospital admission and a history of suicide attempts independently predicted death by suicide.

**Conclusions:**

Our findings suggest there is a high mortality and suicide rate in persons with schizophrenia in rural China, with different predictive factors for mortality and suicide. It is important to develop culture-specific, demographically tailored and community-based mental healthcare and to strengthen family intervention to improve the long-term outcome of persons with schizophrenia.

Over recent decades, evidence has consistently documented markedly elevated worldwide mortality in persons with schizophrenia.^1–3^ One large US study reported that, compared with the general population, people with schizophrenia have 3.5 times higher risk of premature death.^4^ Life expectancy has been estimated to be 19 and 16 years shorter for men and women with schizophrenia respectively.^5^ One recent systematic review suggested that the gap in mortality rates between people with schizophrenia and the general population has been increasing, widening from 2.2-fold in pre-1970s’ to 3.0-fold in post-1970s’ studies.^6^ Notably, suicide accounted for around a quarter of unnatural deaths, especially among males.^4,7^ A meta-analysis estimated that the risk of suicide was approximately 13 times higher for persons with schizophrenia compared with the general population.^8^ Approximately 5% of persons with schizophrenia die by suicide, with elevated rates near illness onset.^9,10^ However, studies examining mortality and suicide among persons with schizophrenia over a long period (i.e. over 20 years) remain scarce. The existing studies are predominantly from Western countries (e.g. Denmark, Norway, the USA, UK and Sweden) and thus may not be generalisable to non-Western countries.^2,6^

China has high rates and unique patterns of schizophrenia and suicide.^11–14^ In the past decade, the prevalence of schizophrenia was around 70–80% higher among females than males and the relative risk of suicide in rural residents with schizophrenia was nearly twofold higher among men than among women. These patterns, however, were different from most countries worldwide.^15^ Compared with urban areas, specifically, the relative risk of suicide in rural China was three to four times higher. Possible explanations for the unique patterns include less availability of mental health services, lack of knowledge about treatment, poverty, dependence on family caregivers, poor family attitudes towards relatives with schizophrenia and the strong stigma of mental illness in rural areas.^16–21^ Nevertheless, trends and predictors for long-term mortality and suicide of persons with schizophrenia in rural China are unknown.

Understanding the long-term trends and factors predicting mortality and suicide among persons with schizophrenia is important for clinicians, especially Chinese clinicians, to identify high-risk patients, improve clinical care and prevent premature death. It can provide information relevant to developing health policies and tailored intervention programmes to increase life expectancy and prevent suicide among this vulnerable population. For public health, this information may facilitate the goal to reduce the health burden of suicide.^2,22^

## Study objectives

The objectives of this study were to (a) explore the 21-year trends of mortality and suicide in persons with schizophrenia in rural China and (b) examine predictors associated with long-term mortality and suicide. This study is the first to explore mortality and suicide trends in persons with schizophrenia in rural China using longitudinal (1994–2015) follow-up.

## Method

### Study population and procedure

The study data were derived from the Chengdu Mental Health Project (CMHP), a prospective longitudinal follow-up study on mental illness and mental health services in Xinjin County, Chengdu, which is a representative middle-income rural county in south-west China.^11,17^ Persons with schizophrenia were identified from a mental health survey in March 1994 among residents aged 15 years or above in six townships of Xinjin County (*n* = 123 572). The six townships were randomly selected from the 12 townships of Xinjin County in 1994.

### Data collection

Data collection procedures in 1994, 2004, 2008 and 2015 have been described in prior studies.^11,20,22^ The time periods of the follow-ups were mainly determined by the availability of funding. Briefly, all persons with schizophrenia (*n* = 510) were identified in 1994 through two steps: (a) screening procedures for psychosis (face-to-face interviews with the head of each household, together with the key informant method) and (b) general psychiatric interview. First, the Psychosis Screening Schedule (PSS) was completed by interviewing the household heads and discussion with village doctors and neighbourhood leaders. Second, once an individual with potential psychosis was identified, a trained psychiatrist conducted a comprehensive general psychiatric interview with that person for further diagnosis. Schizophrenia was diagnosed using ICD-10 diagnostic criteria; diagnostic reliability had been established among the trained research interviewers, who were psychiatrists with more than 5 years of clinical experience. All individuals diagnosed with schizophrenia were followed up in 2004, 2008 and 2015. During the follow-ups, at least one informant familiar with the individual's life and circumstances and/or the cohort members themselves were interviewed. For those who had died, at least one informant familiar with the dead person or the next of kin was interviewed. Of the 1994 sample (*n* = 510), we followed up and interviewed 98.0% (*n* = 500 participants and/or their key informants) 10 years later (May 2004), 95.9% (*n* = 489) 14 years later (June 2008) and 86.5% (*n* = 441) 21 years later (September 2015). The study was approved by the Committee on Human Research Subjects at West China Medical School, Sichuan University and University of Hong Kong and all participants gave informed consent at each stage of the study.

### Measurement

The principle assessment tools included the standardised assessment of the Present State Examination (PSE)^20,22,23^ in the baseline investigation in 1994. In the event of a disagreement, a team of research psychiatrists with more than 10 years of clinical experience reviewed the case to establish the final diagnosis. Since the 2004 follow-up, informants were interviewed by trained psychiatrists using the Patients Follow-up Schedule (PFS)^12^ for about 50 min. The PFS collected information on demographic characteristics, causes and time of death, treatment and social support. For all participants, medical and psychiatric treatment records were also obtained from hospitals, village doctors’ clinics and traditional healers.

#### Mortality and suicide

The analysis outcomes are mortality and suicide, measured by the number of all-cause deaths and suicides collected by interviewers using the PFS in 2004, 2008 and 2015. Interviewers were clinically experienced psychiatrists trained to ensure good reliability of the PFS. Consistent with the previous study,^24^ internal consistency reliabilities for the PFS were adequate (kappa values between pairs of interviewers ranged from 0.76 to 1.00). After the administration of the PFS, two attending psychiatrists reviewed all completed assessments obtained during the interviews. Inconsistent results were presented and discussed in a meeting of all investigators. The classification of each death as due to suicide or to other causes (accident and natural causes) represented the consensus opinion of interviewers and independent researchers. Information from the death certification and suicide note, where applicable, was also obtained.

#### Sociodemographic and illness-related covariates

Sociodemographic (i.e. age, gender, educational level, family size, marital status, family economic status), illness-related (i.e. age at first onset, duration of illness, present mental state, family history of psychosis, prior suicide attempts), treatment-related (i.e. hospital admissions, medication with antipsychotic drugs) and family support status (i.e. family attitude (poor/maltreated versus normal), availability of family care) in 1994 were controlled on theoretical grounds and on the basis of work in mortality and suicide among people with severe mental illness, including schizophrenia.^3,14,25^ Missing data were not replaced by imputation or other methods.

### Statistical analysis

Descriptive analyses were used to investigate the sociodemographic, illness-related and treatment-related characteristics of participants, stratified by gender. Chi-squared (*χ*^2^) analyses or Fisher's exact test for categorical variables and the *t*-test or the Mann–Whitney test for continuous variables were used to assess statistical differences. Kaplan–Meier analysis was performed to estimate time-to-event (all-cause mortality, suicide) distributions and groups were compared using the log-rank test. We calculated life expectancy as the median survival time from baseline by which half of the cohort had died. We used a cause-deleted life table analysis to estimate gains in the number of life-years attributable to reducing suicide deaths, addressing the burden of suicide. Adjusted (gender adjusted for age, age adjusted for all other variables) mortality rates were calculated for suicides, death due to other causes and all-cause mortality from 1994 to 2015. Cox proportional hazards models with backward stepwise likelihood ratio regression were used to estimate possible predictors of mortality and suicide. Hazard ratios (HRs) with 95% confidence intervals (CIs) were provided to account for time at risk. All analyses were done using SPSS software (version 24.0) for Windows.

## Results

### Sociodemographic characteristics and illness-related factors at baseline

At baseline in 1994, 510 persons with schizophrenia (46.5% male, 53.5% female) were recruited (Table 1). Compared with male participants, females reported higher rates of being married (92.7%), having contact with family caregivers (90.8%), better family economic status (49.5%), older age (mean 46.5 years, s.d. = 15.5), older age at first onset (mean 32.3 years, s.d. = 12.5) and higher number of family members (mean 3.7, s.d. = 1.3). No significant differences were observed for other variables (Table 1).

**Table 1:** Sociodemographic characteristics and mental health status of participants with schizophrenia in 1994

	Male (*n =* 237; 46.5%)	Female (*n* = 273; 53.5%)	Total (*n* = 510; 100%)
Educational level (middle school and above), *n* (%)	69 (29.1)	69 (25.3)	138 (27.1)
Married, *n* (%)	115 (48.5)	253 (92.7)***	368 (72.2)
Family history of psychosis (positive), *n* (%)	69 (29.1)	76 (27.8)	145 (28.4)
With family caregivers, *n* (%)	172 (72.6)	248 (90.8)***	420 (82.4)
Family economic status (below average), *n* (%)	143 (60.3)	135 (49.5)*	278 (54.5)
Suicide attempts, *n* (%)^a^	14 (5.9)	23 (8.4)	37 (7.3)
Previous hospital admission	56 (23.6)	54 (19.8)	110 (21.6)
Never treated	81 (34.2)	75 (27.5)	156 (30.6)
Antipsychotic drugs^b^	156 (65.8)	198 (72.5)	354 (69.4)
Current mental status			
Complete remission	58 (24.5)	67 (24.5)	125 (24.5)
Partial remission	26 (11.0)	42 (15.4)	68 (13.3)
Marked symptoms and deterioration	153 (64.6)	164 (60.1)	317 (62.2)
Age, years: mean (s.d.)	42.4 (15.1)	46.5 (15.5)**	44.6 (15.5)
Age at first onset, years: mean (s.d.)	29.6 (13.5)	32.3 (12.5)*	31.0 (13.0)
Duration of illness, years: mean (s.d.)	11.7 (10.6)	13.1 (11.8)	12.5 (11.3)
Number of family members, mean (s.d.)	3.1 (1.6)	3.7 (1.3)***	3.4 (1.5)

a. Suicide attempts are described as frequency and percentage here, but included in the regression model as a binary variable (1 = yes).

b. Frequency and percentage of antipsychotic drug use among participants.

*P < 0.05, **P < 0.01, ***P < 0.001.

### Mortality and suicide in 2015

During the 21 years, a total of 196 (38.4%) participants died, among whom 27 (13.8%) died by suicide and 169 (85.2%) died due to other causes (Table 2). The age-adjusted rates for all-cause mortality were 163.5 (95% CI 116.4–210.6) per 100 000 for the men and 153.0 (95% CI 110.3–195.7) per 100 000 for the women. There were 19 deaths by suicide among the men (age-adjusted rates 30.8 per 100 000) and 8 among the women (age-adjusted rates 13.0 per 100 000, *P* < 0.001). Moreover, participants who had ever used antipsychotic drugs and had no previous hospital admissions had high adjusted rates for all-cause mortality, suicide and death due to other causes.

**Table 2:** Mortality and suicide among participants with schizophrenia in 2015

	Suicide		Death due to other causes		All-cause mortality	
	Observed (*n* = 27), *n*	Adjusted rate (95% CI)^a^	Observed (*n* = 169), *n*	Adjusted rate (95% CI)	Observed (*n* = 196), *n*	Adjusted rate (95% CI)
Gender						
Male	19	30.8 (24.9–36.7)	80	132.7 (89.5–175.9)	99	163.5 (116.4–210.6)
Female	8	13.0 (9.0–17.0)	89	141.6 (100.2–183.0)	97	153.0 (110.3–195.7)
Age group, years						
15–29	11	40.5 (31.2–49.9)	9	33.3 (24.3–42.3)	20	73.6 (65.2–82.0)
30–44	8	20.2 (13.8–26.6)	33	83.4 (77.5–89.3)	41	103.6 (55.3–151.9)
45–59	6	18.6 (12.8–24.4)	63	191.8 (133.3–250.3)	69	210.4 (149.8–271.0)
≥60	2	8.9 (2.5–15.3)	64	259.0 (161.1–356.9)	66	267.9 (169.0–366.8)
Treatment-related factors						
Antipsychotic drugs^b^						
Yes	20	16.2 (12.4–20.0)	110	89.0 (85.7–92.3)	130	105.2 (73.2–137.2)
No	7	5.7 (2.1–9.3)	59	47.8 (40.0–55.6)	66	53.4 (45.6–61.2)
Hospital admission^c^						
Yes	6	4.9 (1.6–8.2)	29	23.5 (16.9–30.1)	35	28.5 (21.5–35.5)
No	21	17.0 (13.1–20.9)	140	113.3 (80.0–146.6)	161	130.3 (95.0–165.6)

a. Adjusted rates: per 100 000 people; confidence intervals for some of the estimates were wide owing to small sample sizes.

b. Participants ever having received treatment with antipsychotic drugs in their illness history.

c. Participants ever having been admitted to hospital in their illness history.

### Predictors of mortality and suicide

**Fig. 1:**
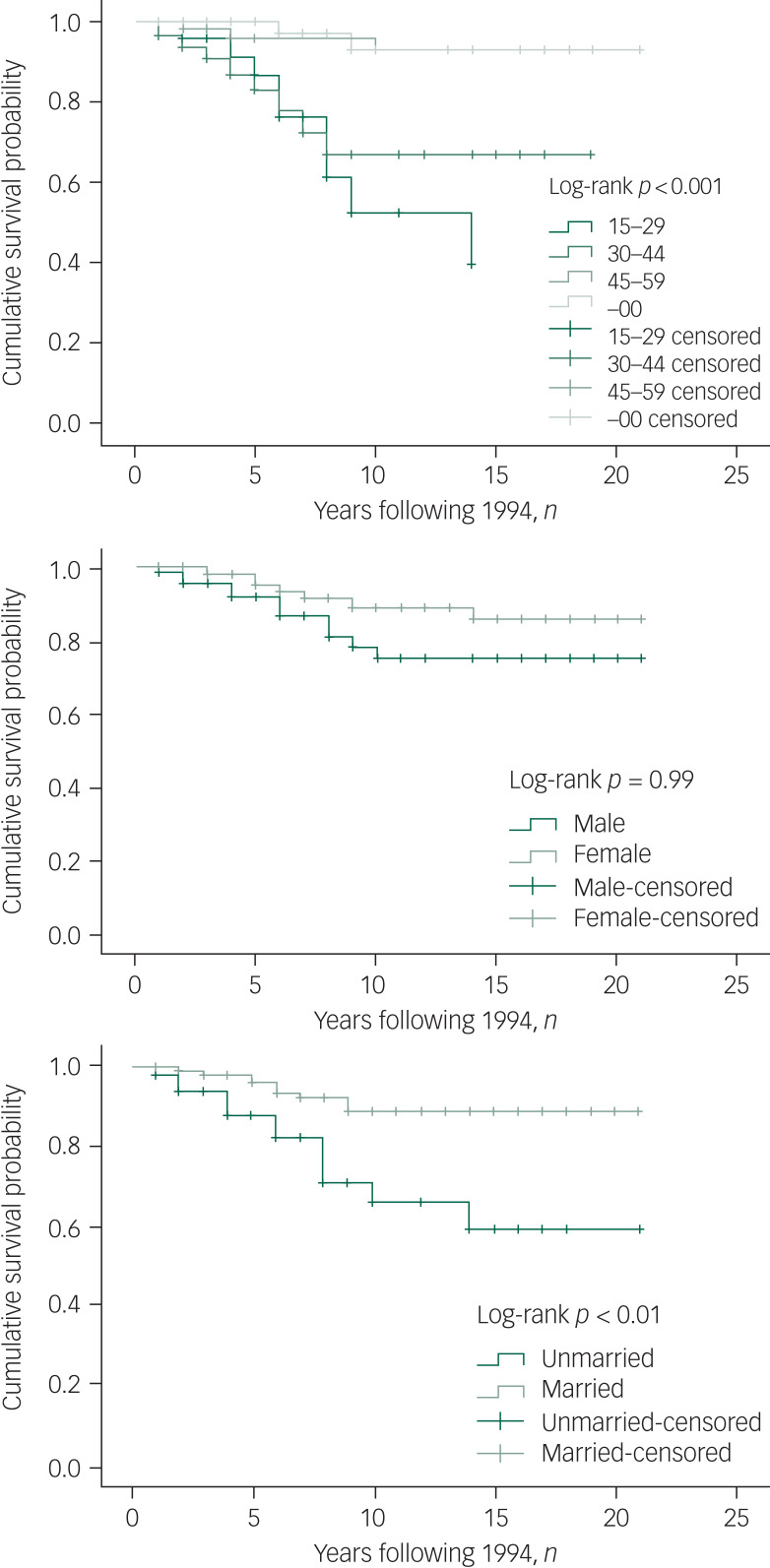
Kaplan–Meier curve for mortality and suicide by age, gender and marital status. Censored, subjects who were lost to follow-up were not counted in the analysis

Figure 1 shows the Kaplan–Meier curves of mortality and suicide for participants by age group, gender and marital status. From 1994 to 2015, the median number of years of survival for participants aged 30–44 (5 years, 95% CI 4.0–6.0) was significantly lower than that for the older subgroups, aged 45–59 (8 years, 95% CI 5.1–10.9) and over 60 years (8 years, 95% CI 6.6–9.4), and the younger subgroup, aged 15–29 (6 years, 95% CI 4.9–6.0; log-rank *P* = 0.011). The median number of years of survival for women was 8 years and for men it was 6 years. Life expectancy among women (58.5 years) was higher than that among men (50.6 years).

For risk of suicide, older participants (≥45 years old) showed significantly longer survival (20.0 years) than participants aged 30–44 (14.4 years) and 15–29 (10.5 years). Males had marginally significantly increased suicide rates compared with females (Fig. 1, *P* = 0.098). Unmarried participants were more likely than the married participants to die by suicide (*P* < 0.01).

For the impact of suicide on total mortality, the results of this study showed that reducing suicide burden would increase the cohort's life expectancy to 61.91 years, under the assumption that the competing causes of death are independent. Thus, if we could eliminate conditions related to suicide, the life-years of persons with schizophrenia would tend to be extended by around 10 years.

**Fig. 2:**
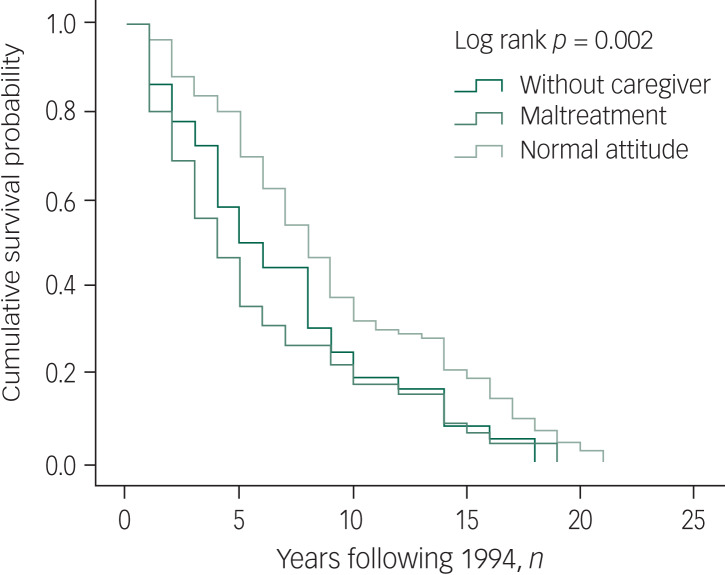
Kaplan–Meier curve for mortality by family attitude.

**Fig. 3:**
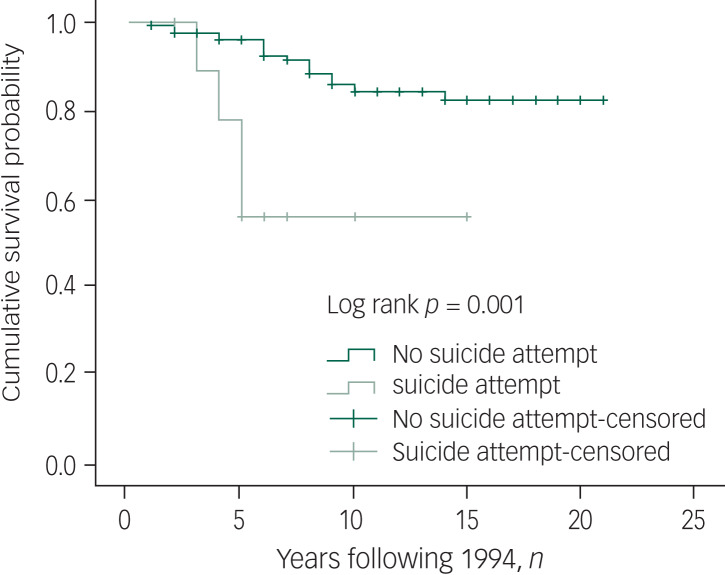
Kaplan–Meier curve for mortality by lifetime suicide attempt. Censored, subjects who were lost to follow-up were not counted in the analysis.

Besides age, gender and marital status as significant predictors of both mortality and suicide, the Kaplan–Meier results also revealed that people being maltreated by family members had shorter survival times (Fig. 2). For suicide, survival time was significantly shorter among people with prior suicide attempts (Fig. 3).

Results of the multivariate Cox regression analysis indicated that older age (HR = 1.02, 95% CI 1.01–1.03), male gender (HR = 1.51, 95% CI 1.12–2.03) and poor family care attitudes towards the individual (HR = 1.40, 95% CI 1.01–1.93) were predictive factors for higher all-cause mortality (Table 3). These factors were also associated with higher likelihood of death due to other causes. Being male (HR = 2.89, 95% CI 1.17–4.01) and having prior suicide attempts (HR = 2.66, 95% CI 1.01–7.26) were risk factors for higher risks of suicide after controlling for other factors. Adjusted Cox regression analysis showed that suicide risk was significantly associated with not being previously admitted to hospital (HR = 6.44, 95% CI 1.59–26.07), whereas being older (HR = 0.95, 95% CI 0.93–0.98) and having a family history of psychosis (HR = 0.36, 95% CI 0.13–0.99) were protective factors against suicide.

**Table 3:** Cox proportional hazards multivariable models predicting mortality and suicide in participants with schizophrenia in 2015

	Suicide		Death due to other causes		All-cause mortality	
	Wald	HR (95% CI)	Wald	HR (95% CI)	Wald	HR (95% CI)
Age (older age, per increasing year)	11.15	0.95 (0.93–0.98)**	20.04	1.03 (1.01–1.04)***	10.87	1.02 (1.01–1.03)***
No previous hospital admission	6.80	6.44 (1.59–26.07)**	1.64	0.77 (0.52–1.15)	1.44	0.79 (0.54–1.16)
Gender (male)	4.27	2.89 (1.07–4.01)*	6.99	1.49 (1.11–2.01)**	7.26	1.51 (1.12–2.03)**
Family history of psychosis	3.86	0.36 (0.13–0.99)*	0.01	1.01 (0.71–1.43)	0.08	1.05 (0.73–1.51)
Prior suicide attempt	3.66	2.66 (1.01–7.26)*	0.31	1.24 (0.58–2.64)	3.09	1.83 (0.93–3.59)
Longer duration of illness (years)	2.63	1.06 (0.99–1.14)	0.30	0.99 (0.96–1.02)	0.12	1.00 (0.99–1.02)
Larger number of family members	2.40	0.79 (0.58–1.07)	1.81	1.07 (0.97–1.19)	1.50	1.07 (0.96–1.18)
Present mental state (with symptoms)	2.12	1.06 (0.82–1.12)	0.90	1.10 (0.91–1.32)	0.10	1.03 (0.84–1.27)
Never treated	1.88	0.56 (0.25–1.28)	0.02	0.97 (0.63–1.48)	0.01	0.99 (0.65–1.51)
Antipsychotic drug use (lifetime)	1.46	4.72 (0.38–58.36)	1.11	0.84 (0.61–1.16)	0.42	0.89 (0.63–1.27)
Education level (no higher than primary school)	1.36	0.60 (0.26–1.41)	0.01	1.02 (0.67–1.55)	0.00	1.00 (0.66–1.51)
Older age at onset (years)	0.54	1.02 (0.97–1.08)	0.62	1.00 (0.98–1.01)	0.01	1.00 (0.97–1.03)
Having caregiver	0.23	0.76 (0.25–2.33)	0.37	0.87 (0.54–1.38)	0.34	0.86 (0.51–1.43)
Poor family attitudes toward patient	0.22	0.73 (0.20–2.71)	5.10	1.45 (1.05–2.03)*	4.18	1.40 (1.01–1.93)*
Married	0.08	1.18 (0.37–3.81)	0.03	1.04 (0.66–1.64)	0.89	1.23 (0.80–1.87)
Family economic status (poor)	0.02	1.09 (0.35–3.35)	0.35	1.12 (0.78–1.60)	0.57	1.14 (0.81–1.60)

HR, hazard ratio.

*P < 0.05, **P < 0.01, ***P < 0.001.

## Discussion

To the best of our knowledge, this is the first population-based, prospective, 21-year follow-up study to examine the trend and predictors of mortality and suicide in persons with schizophrenia, and the first to examine this in China. Findings of this study suggest lower life expectancy among men and higher mortality but lower suicide rates among older adults. In particular, in rural China, poor family attitudes towards relatives with schizophrenia were identified as a specific risk for all-cause mortality, and having not previous hospital admissions and having a history of prior suicide attempts predicted death by suicide.

### Long-term trends of mortality and suicide among persons with schizophrenia

Among the general population in China, life expectancy was 69.7 years for the total population in 1994, with women having longer life expectancy (71.9 years, mortality rate 104.72 per 1000) than men (67.7 years, mortality rate 148.53 per 1000).^26,27^ Between 1994 and 2015, 38.4% of our cohort of people with schizophrenia died. The life expectancy of our participants was 58.5 years for women and 50.6 years for men, which was 13.4 and 17.1 years shorter than that for women and men in the general population in China during the same time period. Although there is a lack of studies examining mortality and suicide in the Chinese population in the 1990s, comparing our results with the weighted average life expectancies in persons with schizophrenia (overall, 64.7 years; female, 67.6 years; male, 59.9 years) in a recent meta-analysis^2^ shows that the life expectancy of persons with schizophrenia in our study was lower by around 9 years. The gender gap in life expectancy identified in our study, where males showed a shorter life expectancy than females, is consistent with prior research.^1,2,5,28^ We also noted that the age at diagnosis in our study is older relative to the literature for both males and females, which may be attributed to unique barriers associated with early detection and treatment in rural China, including poverty, homelessness, lack of health insurance, lack of informed choices of treatment and stigma associated with mental illness.^1–3^

The suicide rate in China was estimated to be 23 per 100 000 people between 1995 and 1999, with the female suicide rate was 25% higher than that among males.^13^ A recent study found that suicide rates in China significantly decreased between 2002 and 2015 (by 16.0% per year until 2006, then by 3.4% per year until 2015), although rates among males were substantially higher than those among females after 2006.^29^ In our study, 13.8% of the all-cause deaths among persons with schizophrenia were attributed to suicide, which is higher than the rate for the general population. The estimated lifetime suicide risk was 5.3% in persons with schizophrenia in this study, which is similar to previous estimates (4.9%).^9^ A cause-deleted life table calculated the gain in life-years after a hypothetical prevention of a suicide, allowing us to measure the significance of the lifetime risk of suicide for mortality among persons with schizophrenia. The result showed that life expectancy of the sample would have significantly increased in the absence of a suicide burden. Our findings extend previous studies and further address the large gap in mortality and suicide rates between persons with schizophrenia and the general population.^13,14,24^

### Predictors of mortality and suicide among persons with schizophrenia

Our results show significant effects of age and gender on mortality and suicide in persons with schizophrenia, and unique predictors for all-cause mortality and suicide. Poor family care attitudes towards participants was found to predict greater mortality and death due to other causes, but not death due to suicide. The effect of poor family attitudes on increasing rates of all-cause mortality and death due to other causes was unique for rural China, where family caregiving and support play a major role in patients’ treatment and care.^30,31^ Evidence shows that having poorer familial attitudes towards persons with schizophrenia might be associated with living without family caregivers, poorer mental status, lower rates of remission, less access to antipsychotic medications and lower social functioning,^31^ which in turn increase the risks of all-cause mortality and death by accidents and other causes. Evidence shows that socioeconomic development has shaped the treatment status of persons with schizophrenia in rural China.^32^ Compared with patients in 1994, persons with schizophrenia in 2015 were more likely to have fewer or no family members and family caregivers, which in turn was associated with never being treated.^32^ Besides, poor family attitudes could increase the internalised stigma in persons with schizophrenia, which might worsen their long-term outcome in rural China.^16^ This is particularly relevant to the Chinese context, given that the majority of persons with schizophrenia (over 90%) are cared for by family caregivers at home.^18,33^

Previous suicide attempts were identified as a predictor of 21-year suicide in this study, which is consistent with previous studies.^16,32^ Not being admitted to hospital was also found to be a predictor of 21-year suicide. Studies in Western countries have suggested that death by suicide among people with severe mental illness peaks during the first year after hospital admission, partly due to the failure of the health system to identify and treat physical diseases during admission for mental disorder.^7,34^ However, many persons with schizophrenia are not admitted in rural China (over 60%), owing to family poverty and poor mental health services, and only some with more severe symptoms are likely to be admitted to hospital.^12,17,18^ Many persons with schizophrenia with aggressive and suicidal behaviour are not treated appropriately (e.g. by admission to hospital, regular medication).^16^ The results of this study show the importance of hospital admission and antipsychotic medication for prevention of suicide in persons with schizophrenia in rural China.^12,18^

Positive family history of psychosis, however, was found to be related to lower suicide rates, which was not observed in a 14-year follow-up study in rural China.^11^ This suggests that family history of psychosis may have significant effect on reducing suicide risk in the longer term. Family history of psychosis was found to be associated with younger age at onset of psychosis, although such difference did not hold for long-term psychiatric symptoms.^11^ It is possible that with longer duration of psychosis, patients and their families gained greater awareness of illness and risks of suicide, which might act as a protective factor against their likelihood of death by suicide.

The results of this study showed that males consistently showed higher rates of mortality and suicide than females, and the gap had been widening over the 21 years, a finding consistent with previous literature in Western countries.^2,6,7,35^ The poor long-term prognosis for men in this study might be explained by the higher rates of being single or divorced, poor family economic status, earlier age at onset of schizophrenia, lack of family caregivers, poor treatment status and higher criminal behaviours.^19,31^ Higher rates of suicide among males than females support the alarming sign of suicide among men in previous literature, including our 14-year follow-up studies.^14,19,36^ This 21-year follow-up study provided further evidence that the large gap in suicides by gender was widening in the long term among rural residents in China.

The results of this study indicated that older persons with schizophrenia had significantly higher all-cause mortality rates than their younger counterparts, although the risk of suicide decreased with age. This study supports the conclusion that age predicts mortality and suicide differently, which is consistent with previous findings in Western countries.^4,7,37^ Differences in mortality and suicide risk estimates are likely related to variations in disease distributions by age: older adults with schizophrenia may have greater exposure to physical diseases, such as cardiovascular disease, diabetes mellitus and respiratory disease.^4,34,38,39^

Suicide, however, was a higher risk among younger adults, a result that is consistent with some prior studies conducted in the Western context.^9,40^ Evidence has shown that persons with schizophrenia often do not receive regular antipsychotic treatment (e.g. clozapine) in rural China.^16^ Severe self-stigma and lack of appropriate treatment during the first onset of illness, especially in rural China where the availability and accessibility of mental health services are limited,^10,13,15^ may be significant factors in suicide among the younger participants.^12,20^ The results of this study further address the importance of developing suicide prevention strategies through community-based mental healthcare to provide earlier diagnosis, antipsychotic treatment, anti-stigma intervention, family support and social functioning rehabilitation.^12,24^

### Implications for policy and services

Our results have implications for healthcare policy and services.^41^ First, the findings can contribute to the understanding of long-term mortality and suicide of persons with schizophrenia, which is important for mental health professionals and policymakers to prevent and reduce mortality and suicide, and promote mental recovery of persons with schizophrenia. Specifically, we identified that men had higher rates of mortality than women, older adults had higher mortality rates and younger adults had higher suicide rates than their counterparts. These findings support efforts to take the demographic differences into account when developing interventions. Males with schizophrenia in rural China comprise a highly vulnerable group who, in addition to psychiatric treatment (e.g. hospital admission, regular antipsychotic treatment), need more support from family, community and society. It is thus important to integrate early diagnosis, treatment, community-based care and family support to improve mental recovery in the rural context. Healthcare practitioners, especially primary healthcare professionals, should be encouraged to strengthen gender-specific mental health intervention by providing targeted resources and treatment when designing intervention programmes in rural China.^19^ Given the serious physical diseases associated with schizophrenia, mental health professionals are encouraged to incorporate physical care in settings treating persons with schizophrenia. Preventions and interventions that aim at improving monitoring of blood pressure and serum glucose level, and managing obesity, hypertension and other cardiovascular risk factors should be further supported to reduce the longevity gap between people with schizophrenia and the general population.^4,6^

Second, it is critical to design culturally and contextually tailored community-based mental health interventions for persons with schizophrenia to reduce the mortality and suicide in rural China. Since poor family attitudes toward persons with schizophrenia were identified as important predictors for all-cause mortality and deaths due to other causes, culturally tailored interventions that target improving family support, reducing stigma of mental illness in patients and their family caregivers, enhancing positive contact and facilitating mental health promotion could be promising steps to increase the effectiveness of interventions.^31,33^

Third, our findings highlight the importance of early interventions for persons with schizophrenia in the efforts to reduce their risks of premature death and death by suicide. Given the limited health resources in rural China, primary healthcare professionals should be trained further to provide community-based mental health services in rural areas. Although duration of illness and never receiving treatment were not risk factors for long-term premature death and suicide in this study, it is crucial to improve early diagnosis, treatment, family-based intervention, community-based mental health services and recovery programmes for persons with schizophrenia in rural China to address the disparity in mortality and suicide.^42^ Given that many persons with schizophrenia do not receive regular antipsychotic treatment in rural China, systematic and regular antipsychotic treatment (e.g. clozapine) should be a crucial aim for reducing suicide among these individuals.^1,14^

### Strengths and limitations

The strengths of this study include the use of three waves of follow-up after a baseline investigation of community samples with schizophrenia, consistent diagnostic criteria over time and high follow-up rates during the 21-year duration.

The limitations include possible recall bias and loss of cases during the long-term follow-up with participants and informants, although such bias may have been reduced by the use of multiple follow-up data sources (i.e. trained psychiatrist interviewers, medical records, death certification). Second, informants might not have reported death by suicide owing to the stigma attached to suicide in the Chinese context. Cross-validation of data collected, however, might reduce such response bias. Third, the number of those within the study who died by suicide was low (*n* = 27). Thus, the results should be interpreted with caution because of the small numbers; furthermore, causality cannot be established owing to lack of a control group. Last, socioeconomic development in China over the 21 years has greatly changed the country's mental health services, including access to treatment and the nature of the treatments received, access to other services and quality of life more generally. Findings of this study may not apply to settings where such changes have not been observed.

## Data Availability

The data are not publicly available because they contain information that could compromise the privacy of research participants.
